# Major environmental and socioeconomic determinants of cutaneous leishmaniasis in Brazil - a systematic literature review

**DOI:** 10.1590/0037-8682-0291-2019

**Published:** 2020-06-01

**Authors:** Lia Puppim Buzanovsky, Manuel José Sanchez-Vazquez, Ana Nilce Silveira Maia-Elkhoury, Guilherme Loureiro Werneck

**Affiliations:** 1 World Health Organization (WHO), Pan American Health Organization (PAHO), Communicable Disease and Environmental Determinants of Health (CDE) / Pan American Center of Foot and Mouth Disease (PANAFTOSA), Department of Epidemiology, Duque de Caxias, RJ, Brazil.; 2 World Health Organization (WHO), Pan American Health Organization (PAHO), Communicable Disease and Environmental Determinants of Health (CDE) Neglected, Tropical and Vector Borne Diseases (VT), Washington, D.C., USA.; 3 Universidade Federal do Rio de Janeiro, Instituto de Estudos em Saúde Coletiva, Rio de Janeiro, RJ, Brasil.

**Keywords:** Cutaneous leishmaniasis, Determinants, Environmental, Socioeconomic, Frequency, Significant association

## Abstract

Cutaneous leishmaniasis (CL) is a zoonotic disease with complex transmission cycle. Some environmental and socioeconomic factors are known to be the major determinants of the transmission process, which are involved in configuring the spatiotemporal patterns and thus can be delimiting. However, the relevance of these socioeconomic and environmental determinants is still not well understood. In this study, we aimed to identify the major environmental and socioeconomic determinants of CL in Brazil by articulating a systematic literature review of studies that are based on this subject. The methodology included a search for studies according to a structured protocol using the scientific platforms, such as Scielo and PubMed. The references of each identified article were who referred to CL determinants were further screened, and so on. We extracted information from 41 articles and the determinants were grouped accordingly. Two measures were evaluated as follows: a) the frequency of citations of the determinants; and b) the proportion of determinants identified as having "significant association in analytical studies" with respect to the total number of determinants analyzed in other analytical studies using the same concept. The analyzed articles covered most of the regions of Brazil and 7 other countries bordering Brazil. We found 43 concepts of determinants. However, the final selection resulted in the identification of 14 major determinants. These results therefore contribute in the identification of major CL determinants and this information can be used to establish strategies for identifying risk prone areas for disease surveillance.

## INTRODUCTION

Cutaneous leishmaniasis (CL) is a non-contagious infectious disease, which is caused by protozoa of the genus *Leishmania* and primarily infect a wide range of animals (wild and possibly domestic hosts^1^), but also humans secondarily. It is a disease transmitted by sandflies - winged hematophagous vectors of the family Psychodidae, genus *Lutzomyia* sp. - that get infected during blood feeding in vertebrate reservoirs and hosts^2^. In Brazil, eleven species of phlebotominae of the genus *Lutzomyia* have been identified as primary vectors. Additionally, epidemiological or the transmission of CL^3^.

The establishment and maintenance of CL transmission cycle can be favored or limited by several environmental and socioeconomic factors, which create barriers for the presence of vectors, reservoirs, and parasites, further preventing or facilitating the occurrence of disease^1^. In the last few decades, many researches have already identified relevant determinants of CL in South America, particularly the environmental ones, for instance in studies regarding the vector ecology in foci areas and other epidemiological studies^4^ ^-^ ^5^ ^,^ ^1^ ^,^ ^6^ ^-^ ^16^.

Human interventions in geographical space and the way this relationship is established are also the major determinants for the maintenance of CL transmission. Leishmaniasis has now expanded beyond its natural ecotypes due to anthropogenic ecological disruptions, which possibly led to an increase in the levels of vector exposure^17^. Therefore, many species have adapted to the modified environmental conditions creating new ecological niches in secondary forests, rural areas, or peridomestic habitats, as well as in non-endemic and urbanized areas^1^ ^,^ ^2^ ^,^ ^17^ ^,^ ^18^. Studies indicate that the construction of large infrastructures, like hydroelectric plants and dams, roads and railways, and implementation of colonization projects in rural areas are highly favorable for CL transmission, as they involve drastic transformation of the local environmental conditions^11^ ^,^ ^19^ ^-^ ^26^.

Similarly, particular socioeconomic settings can also influence the exposure of population to the risk of contracting CL, mainly due to migration, urbanization, and loss of biodiversity, which is associated with deforestation process¹. These changes facilitate the increase in exposure of humans and domestic animals to sandflies^23^ ^,^ ^27^ ^-^ ^28^. Socially organized, integrated, and deeply unequal geographic space determines the occurrence of endemic diseases and their distribution^29^.

In Brazil, CL is distributed across all the regions, but with different prevalences^30^ and great diversity at local level. The dynamics of local variation in CL transmission is related to the diversity of parasitic species, vectors, and reservoirs, various environmental and socioeconomic determinants of land use, revealing different epidemiological patterns of occurrence and dispersion of disease^31^ ^-^ ^34^.

The National Program for the Control and Monitoring of Leishmaniasis in Brazil have described in the surveillance guides the epidemiological patterns based on environmental and socioeconomic determinants as follows: wild pattern in an area of ​​primary vegetation, where the disease is exclusively characterized as zoonosis of wild animals; occupational and leisure pattern associated with the disorderly exploitation of the forest and deforestation for different purposes, including military trainings and ecotourism; rural and periurban pattern in areas of old colonization, related to the migration process, occupation of slopes and agglomerates in urban centers always associated to secondary or residual forests^35^ ^-^ ^37^.

In short, there is a consensus among the experts of leishmaniasis that the environmental and socioeconomic factors might influence CL transmission in Brazil. There is, however, a lack of understanding regarding what are these main determinants that influence CL transmission and their relative significance. In this study, we aimed to identify the main environmental and socioeconomic determinants related to CL occurrence and transmission in Brazil by articulating a systematic literature review.

## METHODS

The methodology of this systematic literature review included a structured protocol to search and identify the studies on zoonoses^38^ ^-^ ^41^ from which the environmental and socioeconomic determinants were further extracted. To determine the distinct relevance of each determinant and select the most relevant ones, a score based on two parameters was proposed by the authors.

### Search protocol and inclusion criteria

This systematic literature review aimed to identify the scientific articles that are focused mainly on environmental and socioeconomic determinants associated with CL transmission in Brazil. For this, the following combinations of words in two different languages (Portuguese and English) were used to perform the search on the DeCS platform (Descriptors in Science and Health: http://decs.bvs.br/P/decsweb2016.htm): “leishmaniose tegumentar” AND “determinantes”; “leishmaniose tegumentar” AND “fatores”; “leishmaniose cutânea” AND “fatores”; “cutaneous leishmaniasis” AND “determinants” AND “Brazil”. The combinations of words in Portuguese and English were used to search for scientific articles on the Scielo and PubMed platforms, respectively.

**FIGURE 1:**
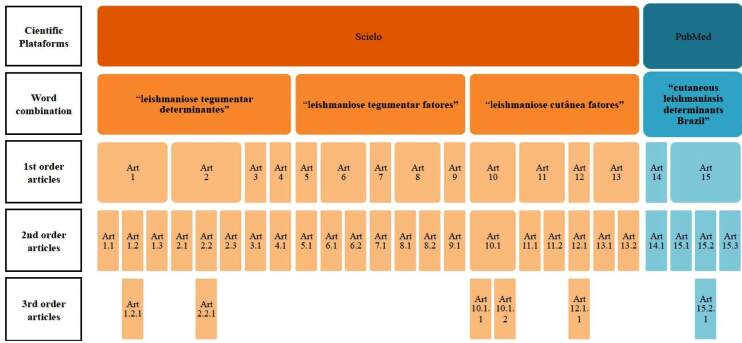
Sample flowchart of the systematic literature review. The numbers indicate the nested level structure implemented within the search. The 1^st^ order articles are the ones that were used in the preparation of this review and are included in the references^5^ ^,^ ^10^ ^,^ ^12^ ^,^ ^13^ ^,^ ^14^ ^,^ ^15^ ^,^ ^16^ ^,^ ^23^ ^,^ ^32^ ^,^ ^43^ ^,^ ^45^ ^,^ ^47^ ^,^ ^50^ ^,^ ^55^ ^,^ ^56^.

Firstly, the articles identified during the primary search (first order articles) were screened. Second, the citations found in these articles of CL determinants cited by other authors were also tracked (second order articles), as shown in Figure 1. However, when new sources were not cited in the screened articles, they were not taken into consideration, and thus, by following this approach, we reached up to the fifth order of articles. This search protocol was developed between July 2016 and June 2017 by a single author.

### Extraction of data from the studies surveyed

Relevant information regarding the studies included in this review that was extracted at this stage: year of publication; type of publication (article, annuals of congress, dissertation, etc.); location of study; origin of the publication (national - Brazil/international); authors; journal; keywords. 

### Classification of environmental and socioeconomic determinants of CL

Environmental and socioeconomic determinants were extracted from the articles identified during the search by the author. Firstly, the determinants were standardized based on the concept, because in some cases similar determinants were denoted with distinct nomenclatures (examples: migratory processes/migration; rainfall/precipitation). This standardization was discussed and agreed among all the researchers who participated in this study.

Thereafter, the determinants were classified based on the type of study, i.e. descriptive or analytical. Subsequently, to assess the level of relevance of each determinant, a score based on two parameters was proposed: 1) "frequency of citations" - presenting the frequency with which the determinants were found in all the studies (either descriptive or analytical), i.e. total number of studies in which the determinant appeared; 2) “proportion with statistical significance” - presenting the proportion of determinants identified with statistically significant association (SA) with the disease in analytical studies (AS), i.e. the number of times a determinant was found associated over the total number of analytical studies in which the determinant was studied. 

Finally, based on the results of this score, the determinants that were equal to or above the 85^th^ percentile for the "frequency of citations" in all the analyzed studies, and equal or above the 85^th^ percentile for the “proportion with statistical significance” in the analytical studies, were considered to exhibit significant relevance. Some cutoff points were tested, and then the results were evaluated by the authors to choose the cutoff that would represent the highest number of important determinants in two categories taken into consideration. The calculations for descriptive statistics were performed using the free statistical software R (R Core Team, 2018) of the Hmisc package^42^.

## RESULTS

### Characterization of the studies included in this review

**FIGURE 2:**
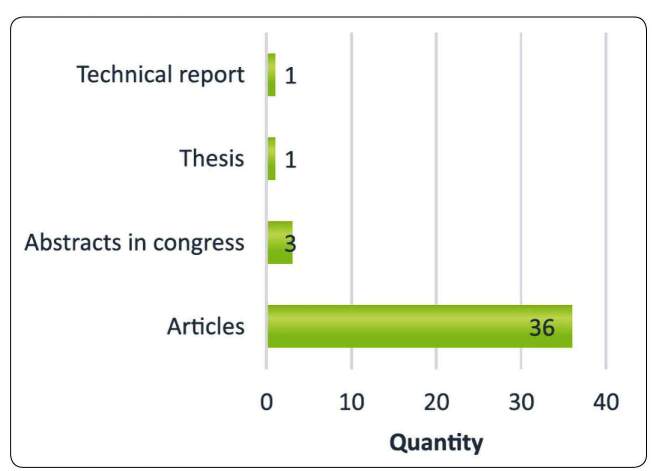
Types of publications identified through the literature review.

The initial search resulted in the identification of 15 first-order scientific articles. After performing the successive screening of these articles until the fifth order, a total of 41 publications were identified, involving 148 authors, and 88 % (36) of these publications were scientific articles (Figure 2). There were 32 publications from national Brazilian publication sources and 9 from international publication sources published from 1981 to 2012.

**FIGURE 3:**
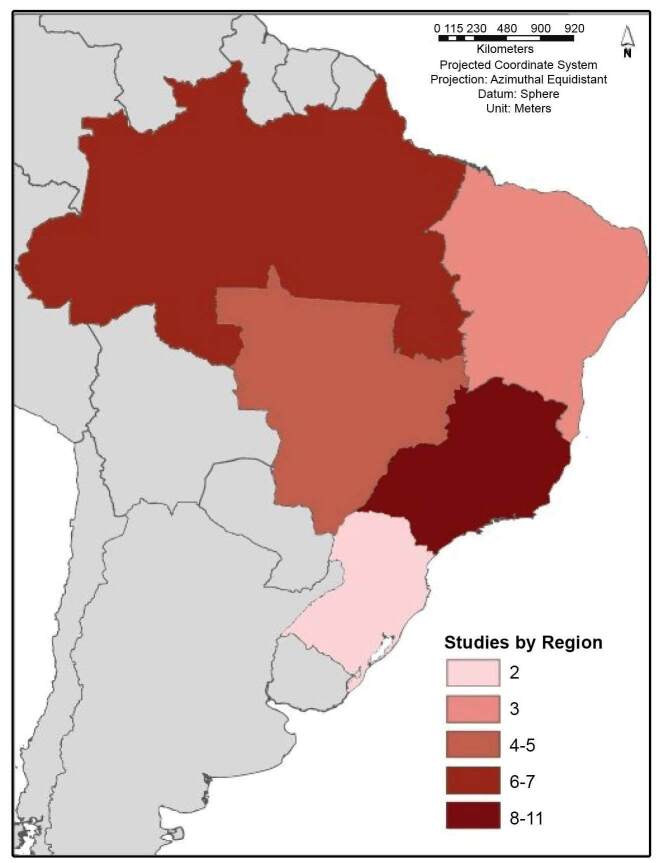
Number of studies corresponding to different regions of Brazil.

All the regions of Brazil were contemplated, with 28 studies that were focused on specific states, including Acre, Amazonas, Bahia, Espírito Santo, Mato Grosso, Mato Grosso do Sul, Minas Gerais, Pará, Paraná, Pernambuco, Rio de Janeiro, and São Paulo. The region that was included in most of the studies was the Southeast region (11), followed by the North (7), Midwest (5), Northeast (3), and South (2) regions (Figure 3). 

Five studies were carried out at the national level in Brazil, and another five studies were focused on other South American countries, which share the borders with Brazil, and were also considered in this study because it is considered that the disease, vector, parasites, as the environmental and socioeconomic determinants are not determined by the administrative borders. Also, three other studies that were carried out at the Regional level (South America), encompassing Brazil were also included. 

### Identification of determinants

A total of 169 environmental and socioeconomic determinants were identified in the primary list that were clustered during the standardization process in 43 concepts of determinants and were highlighted according to the form or type of studies in which they were identified (Table 1). 

**TABLE 1:** Frequency of studies based on the environmental and socioeconomic determinants that were identified.

Groups of environmental and socioeconomic determinants				
Groups	With SA in AS	Total in AS	Total in DS	Total of JM
Sanitation	1	2	9	6
Agricultural and livestock	1	6	8	12
Agricultural Development Projects	0	0	1	6
Agroindustry	0	2	0	2
Air humidity	1	8	1	8
Altitude	0	3	0	1
Animal production	2	6	1	2
Armed conflicts	0	0	0	2
Bioclimatic zones	0	0	0	1
Climate phenomena	0	1	0	5
Construction of large infrastructures	0	0	0	12
Cultural habits that cause exposure	5	6	0	10
Deforestation	1	2	1	21
Degree of urbanization	1	1	1	5
Forestry explorations	0	0	0	2
House near nature	0	2	5	9
Human development	1	2	0	0
Income	2	4	2	4
Land use	0	2	2	3
Leisure and tourism activities	0	1	0	4
Life expectancy	0	1	0	0
Migration processes	0	0	0	5
Mining activities	1	1	0	3
Natural ecological changes	0	0	0	3
Non-rural activities	0	1	0	0
Population concentration	1	3	1	4
Presence of domestic animals	2	5	9	3
Presence of forests	1	6	3	23
Presence of reservoirs	0	0	3	4
Presence of vectors	2	5	3	8
Presence of water	0	1	0	1
Professional occupation	3	3	9	22
Protected areas	0	1	0	0
Rainfall	2	9	2	3
Relief	0	4	0	2
Rural area	1	3	3	18
Scholarity	0	6	0	0
Solar radiation	0	1	0	1
Temperature	1	17	1	4
Type of soil	0	1	1	2
Types of vegetation	0	3	1	15
Urban and periurban area	1	3	2	5
Rural population	2	4	0	0

The results for the final selection of determinants by "frequency of citations" in descriptive studies indicate a median of 9.49 and an interquartile range between 3.75 and 16 citations. The 85^th^ percentile that marked the cut-off (equal or above) to select the determinants base on this parameter was set at 18. Conversely, the results for the final selection of determinants by “proportion with statistical significance” in analytical studies indicate a median of 0.324 and an interquartile range between 0 and 0.40. The 85^th^ percentile that marked the cut-off (equal or above) to select the determinants based on this parameter was set at 0.5. 

As a result, the determinants selected by “frequency of citations” include professional occupation, presence of forests, agriculture and livestock activities, deforestation, rural areas, temperature, and types of vegetation. The determinants selected by “proportion with statistical significance” include degree of urbanization, mining activities, professional occupation, cultural habits, access to basic public services and sanitation, deforestation, human development, income, and urban/rural population. The determinants, namely occupation and deforestation, have surpassed in the two classification categories simultaneously (“frequency” and “proportion with statistical significance”) and the final selection resulted in a total of 14 most relevant determinants (Table 2).

**TABLE 2:** Determinants selected by using “proportion with statistical significance” and “frequency of citations”.

Determinants selected by “proportion with statistical significance”		Determinants selected by “frequency of citations”	
Groups	Proportion SA/AS	Groups	Frequency
Degree of urbanization	1	Professional occupation	34
Mining activities	1	Presence of forests	32
Professional occupation	1	Agricultural and livestock activities	26
Cultural habits that cause exposure	0.83	Rural areas	24
Deforestation	0.5	Deforestation	24
Human development	0.5	Temperature	22
Income	0.5	Types of vegetation	19
Rural population	0.5		
Access to basic public services and sanitation	0.5		

## DISCUSSION

In this study, we identified the most relevant environmental and socioeconomic determinants related to the occurrence and transmission of CL in Brazil by articulating a systematic literature review, which utilized diverse types of studies, from both national (covering all the regions in Brazil) and international sources.

In previous studies, authors have associated the labor activities performed by the individuals as a major determinant for the occurrence of CL, and therefore occupational risk was identified more frequently as a determinant in this systematic literature review. The most cited occupations that favor the transmission of CL were related to forestry activities^1^ ^,^ ^2^ ^,^ ^6^ ^,^ ^8^ ^,^ ^26^ ^,^ ^28^ ^,^ ^43^, including extraction (e.g. extraction of latex, collection of brazil nuts, açaí)^1^ ^,^ ^23^ ^,^ ^26^ and military training activities^1^ ^,^ ^17^ ^,^ ^25^ ^,^ ^26^ ^,^ ^40^ ^,^ ^41^. Since these activities are labor driven and promote incursions in the rainforest areas, they contribute significantly to the exposure of this group of workers to the risk of infection. Similarly, other practices that can facilitate the exposure of individuals to CL, including fishing^6^ ^,^ ^15^, hunting^1^ ^,^ ^6^ ^,^ ^46^ ^,^ ^47^, catch firewood^47^, forest incursion habits^4^ ^,^ ^22^ ^,^ ^46^ ^,^ ^48^ explains the appearance of cultural habits as an important determinant in this study. 

Consequently, areas with agricultural activities, such as cultivation of banana, cocoa, coffee, sugar cane, and fruit crops in general, as well as animal production of chickens, pigs and horses, were associated with the risk of occurrence and transmission of CL^6^ ^,^ ^12^ ^,^ ^15^ ^,^ ^16^ ^,^ ^20^ ^,^ ^24^ ^,^ ^28^, as both are related to facilitate conditions for the presence or situation of contact with the vectors. Moreover, due to the same reason, some studies determined the proximity of houses from agriculture plantations as a risk factor^10^ ^,^ ^23^ ^,^ ^28^ ^,^ ^43^ ^,^ ^47^. Besides, being part of rural population^13^and residing in a rural area, for instance, in specific places such as settlements or quilombola (communities of descendants of African slaves who have their territory demarcated by law in Brazil and practice subsistence agriculture) areas, implies an increased risk. Therefore, this was consistently found in the literature as a CL determinant when compared to living in urban/peri urban areas^1^ ^,^ ^6^ ^,^ ^13^ ^,^ ^20^ ^,^ ^21^ ^,^ ^22^ ^,^ ^24^ ^,^ ^49^ ^,^ ^50^. Living in these places or to be part of such population implies higher risk of contact with natural environment where the vectors and reservoirs are found more frequently.

The higher average annual temperature is also considered as an important risk factor^5^ ^,^ ^6^ ^,^ ^7^ ^,^ ^8^ ^,^ ^9^ ^,^ ^10^ ^,^ ^12^ ^,^ ^13^ ^,^ ^26^ ^,^ ^14^ ^,^ ^48^ ^,^ ^55^ because it might favor the presence of vectors in tropical forests and is more conducive to the presence of vectors. In general, it is assumed by researchers that the closer an individual is to the forest or dense vegetation areas, higher are the risk of contracting infections. The main environmental determinants associated with favorable conditions for the presence of vectors and wild reservoirs of CL were the presence of forests^12^ ^,^ ^15^ ^,^ ^21^ ^,^ ^22^ ^,^ ^23^ ^,^ ^24^ ^,^ ^42^ ^,^ ^44^ ^,^ ^47^ ^,^ ^49^ ^,^ ^50^ ^,^ ^52^ and certain types of vegetation^7^ ^,^ ^15^ ^,^ ^16^ ^,^ ^26^ ^,^ ^48^ ^,^ ^54^. Likewise, sudden intervention with the environment with intense contact between the agents involved in the transmission cycle of CL and the human, such as mining activities^21^ ^,^ ^23^ ^,^ ^51^ and deforestation^1^ ^,^ ^4^ ^,^ ^6^ ^,^ ^7^ ^,^ ^12^ ^,^ ^15^ ^,^ ^20^ ^,^ ^21^ ^,^ ^22^ ^,^ ^23^ ^,^ ^24^ ^,^ ^25^ ^,^ ^26^ ^,^ ^43^ ^,^ ^46^ ^,^ ^48^ ^,^ ^53^ ^,^ ^54^were also referred as important determinants. 

To identify the populations or regions under high risk, studies have highlighted determinants, such as economic productivity, human development, and income^12^ ^,^ ^13^ ^,^ ^24^ ^,^ ^56^. With regard to economic productivity, the highest risk is imposed in the regions with a primary sector-based economy (e.g., agriculture, mining, fishing, livestock, plant extractive and hunting) than in secondary (industry) and tertiary (services). CL is a neglected disease that is considered to affect mainly the vulnerable populations, and thus it is assumed that low-income and undeveloped populations are at higher risk. Similarly, determinants related to infrastructure, such as the degree of urbanization^12^ ^,^ ^13^ ^,^ ^15^ ^,^ ^22^ ^,^ ^24^ ^,^ ^26^ and access to basic public services and sanitation^9^ ^,^ ^14^ ^,^ ^16^ ^,^ ^23^ ^,^ ^24^ ^,^ ^26^ ^,^ ^47^ ^,^ ^49^ were evaluated as significantly relevant, further assuming that higher the precariousness, which mean lower degree of urbanization, sanitation and access to basic public services, higher is the risk.

To generate the list of major determinants, the chosen parameter, namely "frequency of citations" and “proportion with statistical significance” exhibit the following purposes: the first parameter represents the emphasis made by researchers and the relevance of each determinant when delimiting and prioritizing what determinants should be evaluated in their respective study. The second parameter aimed to identify the determinants based on quantitative evidence regarding its association with CL occurrence. 

In this study, we cannot conclude that the results found are certain to indicate the major socioeconomic and environmental determinants of CL, since there are several limitations. The results can be influenced by the time period in which the review was conducted, so we would recommend that the methodology should be replicated every ten years in order to update the results. 

In addition, researchers might have a preference towards these determinants, but there might be others that are yet to be explored. Likewise, determinants with limited research might still be important and they are just being neglected due to several reasons including: (1) lack of previous knowledge; (2) high cost to be measured in practice (e.g. identification of tree species that would require botanical assessment at the local level or high-resolution satellite imagery); (3) difficulty in operationalizing concepts for empirical research, such as social inequality and social vulnerability. Also, it is important to consider the fact that if the association is not significant, it may reflect only statistical caveats, such as small sample size. By addressing these limitations, we believe that the methodology used is both sensitive and robust, since it encompasses highly cited determinants together with those that have exhibited significant association in analytical studies. 

Finally, the sole intention behind conducting this systematic literature review was to contribute in a better epidemiological characterization of the disease in order to establish new strategies for the identification of risk prone areas, and thus, to improve CL surveillance in different regions of Brazil. This methodology can also be applied to identify the environmental and socioeconomic determinants to another zoonosis, in order to support risk-based surveillance.
